# Leflunomide increases the risk of silent liver fibrosis in patients with rheumatoid arthritis receiving methotrexate

**DOI:** 10.1186/ar4075

**Published:** 2012-10-29

**Authors:** Sang-Won Lee, Hee-Jin Park, Beom Kyung Kim, Kwang-Hyub Han, Soo-Kon Lee, Seung Up Kim, Yong-Beom Park

**Affiliations:** 1 Department of Internal Medicine, Yonsei University College of Medicine, 50 Yonsei-ro, Seodaemun-gu Seoul, 120-752, Korea; 2 Institute for Immunology and Immunological Diseases, Yonsei University College of Medicine,; 50 Yonsei-ro, Seodaemun-gu; Seoul, 120-752, Korea; 3 Department of Medical Sciences, Yonsei University College of Medicine, 50 Yonsei-ro, Seodaemun-gu; Seoul, 120-752, Korea; 4 Institute of Gastroenterology, Yonsei University College of Medicine, 50 Yonsei-ro, Seodaemun-gu; Seoul, 120-752, Korea; 5 Liver Cirrhosis Clinical Research Center, Yonsei University College of Medicine, 50 Yonsei-ro, Seodaemun-gu; Seoul, 120-752, Korea

## Abstract

**Introduction:**

We identified silent liver fibrosis in patients with rheumatoid arthritis (RA) using transient elastography, and investigated medication that correlated with abnormal liver stiffness measurement (LSM) values.

**Methods:**

We consecutively enrolled 105 patients with RA taking methotrexate over 24 weeks with normal liver functions and no history of underlying chronic liver disease. Blood tests were performed, and body mass index and metabolic syndrome were assessed. We checked LSM values, and adopted 5.3 kPa as the cutoff for abnormal LSM values. The cumulative doses of medications including methotrexate, leflunomide, sulfasalazine, hydroxychloroquine, prednisolone, meloxicam, and celecoxib were calculated.

**Results:**

The median age of patients (20 men and 85 women) was 52.4 years. The median LSM value was 4.7 kPa and 24 (22.9%) patients had abnormal LSM values. Gamma-glutamyltranspeptidase levels and the cumulative doses of leflunomide and prednisolone significantly correlated with LSM values (*P*<0.05). The cumulative dose of leflunomide, but not methotrexate, was significantly higher in patients with abnormal LSM values than that in patients with normal LSM values (*P *= 0.008). When RA patients receiving leflunomide plus methotrexate were classified into two groups according to the optimal cutoff cumulative dose of leflunomide (19,170 mg), abnormal LSM values were more frequently identified in patients with high cumulative dose of leflunomide (odds ratio, 12.750; *P*<0.001).

**Conclusions:**

The cumulative dose of leflunomide was the only independent predictor of abnormal LSM values in patients with RA who had received methotrexate for more than six months.

## Introduction

Rheumatoid arthritis (RA) is characterized by synovial inflammation in multiple joints and irreversible joint destruction in the absence of adequate treatment [1,2]. Among various disease-modifying anti-rheumatic drugs (DMARDs), methotrexate (MTX) has been the most widely used of these for RA. Moreover, new biological agents, such as TNF-α blockade and anti-CD20 monoclonal antibody, need a combination treatment with MTX to reduce the formation of neutralizing antibodies that can diminish the therapeutic efficacy [3].

Thus, unless patients have systemic conditions that make MTX treatment unfeasible, such as liver or interstitial lung disease, MTX is usually administered to most RA patients, either alone or with other DMARDs [4,5]. Despite their potent efficiency, long-term use of MTX can induce serious adverse events such as hepatitis [6], although the development of liver cirrhosis due to MTX use has rarely been reported [7]. Other DMARDs or non-steroidal anti-inflammatory drugs (NSAIDs) may not be free from concerns over these adverse effects. Particularly, leflunomide, which is newly developed and is often used in combination with MTX, has been reported to significantly increase the risk of liver toxicity potentially elevating liver enzyme levels, or inducing other serious diseases such as liver fibrosis [8,9].

From a clinical point of view, physicians may stop DMARDs or reduce their doses, when the levels of liver enzymes are highly elevated. However, drug-induced liver fibrosis can sometimes progress without changes of enzyme levels, or of structure on ultrasonography. In these cases, only liver biopsy can detect silent liver fibrosis, but it cannot be performed in all patients taking DMARDs, because it is invasive and unethical.

Recently, non-invasive liver stiffness measurement (LSM) using transient elastography (FibroScan^®^, EchoSens, Paris, France) was introduced to assess the severity of liver fibrosis, and to screen the normal population for identifying people potentially at risk of underlying chronic liver disease [10-12]. So far, a few studies using transient elastography have shown a relationship between MTX use and liver fibrosis, but the association still remains controversial [13-15]. Furthermore, the combined effect of MTX and other DMARDs on liver fibrosis has not been described. Hence, in the present study, we assessed the correlation of the dose of MTX and silent liver fibrosis and investigated medication that correlated with abnormal liver stiffness measurement (LSM) using transient elastography in RA patients receiving MTX.

## Materials and methods

### Patients

We consecutively enrolled 150 patients with RA to this study from October 2011 to January 2012 according to the initial inclusion criteria as follows: (1) patients diagnosed at the Division of Rheumatology, Severance hospital, Yonsei University College of Medicine, with RA based on the American College of Rheumatology 1987 revised criteria [16]; (2) those who had received MTX and/or other DMARDs over 24 weeks; (3) those who had no history of chronic liver diseases, such as viral hepatitis or structural abnormalities identified in the 10th revised International Classification of Diseases (ICD-10); (4) those who had never received medication for liver diseases under the Korean Drug Utilization Review (DUR) system; and (5) those who gave informed consent for their participation. The majority of patients (94.3%) took blood tests on the same day they underwent LSM and ultrasonography, while six patients had blood tests up to 2 weeks earlier.

The additional inclusion criteria based on the results of laboratory tests or LSM and ultrasonography included: normal ranges of platelet count (> 150,000/mm3), aspartate aminotransferase (AST) (≤ 40 IU/L), alanine aminotransferase (ALT) (≤ 40 IU/L), total bilirubin (≤ 1.2 mg/dL), serum albumin (≥ 3.5 mg/dL), gamma-glutamyltranspeptidase (GGT) (≤ 54 IU/L), alkaline phosphatase (ALP) (≤ 115 IU/L), prothrombin time (≤ 1.16 international normalized ratio [INR]), successful or reliable LSM, and normal structure on ultrasonography.

**Figure 1:**
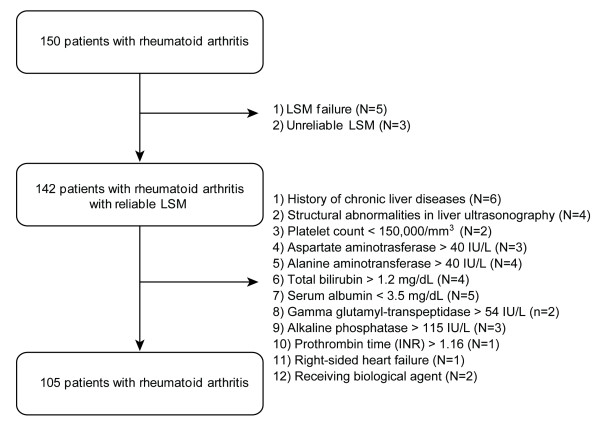
**Selection of the study population**. LSM, liver stiffness measurement; INR, international normalized ratio.

Among 150 patients who were recruited, eight (5.3%) were excluded due to LSM failure (*n *= 5) or unreliable LSM (*n *= 3) (Figure 1). Of those with reliable LSM, 37 patients were further excluded based on our exclusion criteria described above (Figure 1). Finally, 105 patients were selected for statistical analysis. This study was approved by the Institutional Review Board of Severance Hospital.

### Demographic and laboratory findings and medications

On the same day as the LSM, height, weight, waist circumference, and blood pressure were also measured to calculate body mass index (BMI) and determine the prevalence of metabolic syndrome. Metabolic syndrome was diagnosed in subjects showing at least three of the five following features: (1) waist circumference > 90 cm in men and > 80 cm in women according to ethnicity; (2) triglycerides ≥ 150 mg/dl (1.7 mmol/L); (3) high density lipoprotein (HDL) cholesterol < 40 mg/dl (1.03 mmol/L) in men and < 50 mg/dl (1.29 mmol/L) in women; (4) blood pressure ≥ 130/85 mmHg and (5) fasting plasma glucose ≥ 110 mg/dl [17]. The laboratory results (as per the aforementioned additional inclusion criteria), were obtained on the day of the LSM. The cumulative doses of medications including MTX, leflunomide, sulfasalazine, hydroxychloroquine, prednisolone, meloxicam, and celecoxib were calculated. The cumulative dose of each medication was defined as the accumulated area under the curve (AUC) from the time of drug initiation to the time of LSM during the interval follow-up days (see Additional file 1).

### Liver stiffness measurement and ultrasonography

LSM was performed by a single experienced independent physician (who had previously performed more than 10,000 examinations) blind to the clinical data of the study population, according to the examination protocol described in previous studies [10-12]. The results were expressed as kilopascals (kPa), and the success rate was calculated as the number of valid measurements divided by the total number of measurements. Only LSM examinations with at least 10 validated measurements and a success rate of at least 60% were considered reliable. The median value of successful measurements was selected as the representative LSM value for that subject, when an interquartile range to median value ratio was less than 0.3. Any LSM that did not satisfy the above conditions was considered unreliable and was excluded from further analysis. We also performed ultrasonography to exclude the patients who had any morphological abnormalities that might affect the LSM results.

### A cutoff value for abnormal LSM

We referred to LSM values which were derived from the most well-designed Asian study that investigated healthy living liver and kidney donors in South Korea (with LSM 5^th ^and 95^th ^percentiles 3.9 to 5.3 kPa) and we adopted 5.3 kPa as the cutoff value for abnormal LSM values, indicating the potential development of silent liver fibrosis [10].

### Statistical analyses

All statistical analyses were conducted using the SPSS package for Windows version 11.5 (SPSS Inc., Chicago, IL, USA). Continuous variables were expressed as mean ± SD or median (range), as appropriate. Significant differences between the two groups according to LSM values above 5.3 using the chi square test, and Fisher's exact test for categorical data, and the Mann-Whitney test was used for continuous variables. The correlation between LSM values and variables was evaluated using univariate Pearson's correlation. Univariate analysis of association between LSM values and each variable was performed using linear regression. The odds ratio (OR) was assessed using multivariate logistic regression of variables with *P*-values less than 0.05 upon univariate analysis. The optimal cutoff value of the cumulative dose of leflunomide for the prediction of abnormal LSM values was extrapolated by calculating the area under the receiver operator characteristic curve (AUROC), and selection to maximize the sum of sensitivity and specificity. In addition, the OR of the cumulative dose of leflunomide for abnormal LSM values was analyzed using contingency tables and the chi square test. *P*-values < 0.05 were considered statistically significant.

## Results

### Baseline characteristics and comparison between patients with and without abnormal LSM values

The baseline characteristics are summarized in Table 1. The median age of the 105 patients (20 men and 85 women) was 54 years. The median BMI was 22.1 kg/m^2^, and six patients (5.7%) had metabolic syndrome. The median disease duration was 140.4 (range, 26 to 739) weeks. The median cumulative doses of MTX and leflunomide were 2,032.5 mg and 7,800.0 mg, respectively. The median LSM value was 4.4 (range, 2.8 to 17.8) kPa. Twenty four of the 105 patients (22.9%) had abnormal LSM values and three patients (2.9%) had LSM values over 8.0 kPa.

**Table 1:** Baseline characteristics in RA patients who were receiving MTX and comparison between patients with and without abnormal liver stiffness measurement (LSM)

	All patients (*n *= 105)	LSM < 5.3 kPa(*n *= 81)	LSM > 5.3 kPa(*n *= 24)	*P*-value
Demographic variables				
Age, years	54.0 (25, 73)	52.5 ± 10.3	52.3 ± 11.8	0.852
Male gender	20 (19.0)	16 (19.8)	4 (16.7)	0.736
Body mass index, kg/m^2^	22.1 (18.1, 27.6)	22.1 ± 2.5	22.3 ± 2.4	0.723
Metabolic syndrome, number patients (%)	6 (5.7)	3 (3.7)	3 (12.5)	0.105
Disease duration, weeks	140.4 (26.1, 739.2)	171.8 ± 123.9	240.5 ± 159	0.059
Laboratory variables				
C-reactive protein, mg/L	1.2 (0, 124.0)	3.1 ± 5.7	6.9 ± 25.1	0.766
Erythrocyte sedimentation rate, mm/hr	33.0 (2, 111)	37.3 ± 22.3	32.4 ± 20.7	0.362
White blood cells, count/mm^3^	5,960.0 (2,390.0, 12,870.0)	6,355.9 ± 2,009.0	5,801.7 ± 1,853.9	0.319
Hemoglobin, g/dL	12.7 (9.9, 16.2)	12.7 ± 1.1	13.0 ± 1.0	0.324
Platelet count, × 1,000/mm^3^	241.0 (134.0, 470.0)	253.0 ± 60.3	250.8 ± 69.0	0.723
Prothrombin time, INR	0.9 (0.8, 1.2)	1.0 ± 0.1	1.0 ± 0.1	0.726
Glucose, mg/dL	85.0 (70.0, 125.0)	87.4 ± 10.7	86.4 ± 5.0	0.652
Blood urea nitrogen, mg/dL	14.4 (0.9, 28.1)	15.2 ± 4.8	14.1 ± 4.6	0.249
Creatinine mg/dL	0.7 (0.5, 1.3)	0.7 ± 0.2	0.7 ± 0.1	0.858
Uric acid, mg/dL	3.8 (1.5, 8.9)	4.0 ± 1.2	4.1 ± 1.8	0.582
Aspartate aminotransferase, IU/L	21.0 (13.0, 34.0)	21.9 ± 5.5	22.4 ± 6.8	0.881
Alanine aminotransferase, IU/L	17.0 (8.0, 39.0)	18.9 ± 7.7	19.0 ± 7.7	0.879
Total protein, mg/dL	6.9 (5.5, 7.9)	7.0 ± 0.4	7.0 ± 0.5	0.730
Serum albumin, mg/dL	4.3 (3.5, 4.9)	4.3 ± 0.3	4.2 ± 0.4	0.815
Total bilirubin, mg/dL	0.6 (0.2, 1.1)	0.6 ± 0.2	0.6 ± 0.2	0.461
Alkaline phosphatase, IU/L	52.0 (38.0, 110.0)	56.4 ± 17.0	54.7 ± 13.1	0.976
Gamma-glutamyltranspeptidase, IU/L	17.0 (5.0, 53.0)	22.6 ± 15.1	26.3 ± 19.1	0.340
Triglyceride, mg/dL	90.0 (26.0, 269.0)	108.9 ± 55.4	92.2 ± 41.4	0.197
High density cholesterol, mg/dL	55.0 (19.0, 86.0)	55.9 ± 10.4	53.2 ± 19.1	0.772
Low density cholesterol, mg/dL	105.4 (52.8, 172.6)	108.8 ± 26.8	107.4 ± 26.8	0.743
Cumulative dose of medication, mg				
Methotrexate (105 patients)	2,032.5 (285.0, 7,800.0)	2,334.0 ± 1,939.9	3,127.9 ± 2,072.7	0.086
Leflunomide (53 patients)	7,800.0 (280.0, 38,520.0)	9,291.1 ± 9,331.8	19,919.3 ± 12,716.8	**0.008**
Sulfasalazine (82 patients)	360,000.0 (30,000.0, 6,028,000.0)	727,778.7 ± 969,150.7	1,093,547.6 ± 1,330,930.2	0.111
Hydroxychloroquine (65 patients)	94,600.0 (4,200.0, 868,000.0)	115,248.0 ± 120,975.1	182,013.3 ± 208,807.9	0.129
Prednisolone (89 patients)	3,015.0 (372.5, 20,212.5)	4,642.1.0 ± 4,196.7	6,756.7.1 ± 6,156.5	0.472
Meloxicam (90 patients)	6,273.8 (210.0, 2,895,000.0)	90,205.0 ± 363,930.2	161,274.6 ± 644,791.4	0.793
Celecoxib (70 patients)	163,000.0 (1,000.0, 1,503,000.0)	218,505.7 ± 253,487.6	316,705.9 ± 313,982.7	0.239
LSM				
LSM, kPa, median (range)	4.4 (2.8, 17.8)	NA	NA	NA
LSM, kPa, median (IQR)	0.14 (0.02, 0.29)	NA	NA	NA
Success rate, %, median (IQR)	100.0 (63.0, 100.0)	NA	NA	NA

Values are expressed as median (range, IQR), number (%), or mean ± SD. INR, international normalized ratio; NA: not applicable.

Twenty-four of the 105 patients (22.9%) had abnormal LSM values when patients were classified into two groups according to the cutoff (5.3 kPa). There were no significant differences between the two groups in disease duration, demographic, or laboratory variables, including liver enzymes. Among medications, the cumulative dose of leflunomide in patients with abnormal LSM values was significantly higher than that in patients with normal LSM values (*P *= 0.008) (Table 1).

### Correlation between LSM and other variables

In the univariate analysis, GGT levels and the cumulative doses of leflunomide and prednisolone significantly correlated with LSM values (*r *= 0.249, *r *= 0.285, and *r *= 0.362, *P *< 0.05 for all), whereas the cumulative dose of MTX showed no significant correlation with LSM values (*P *= 0.273) (Additional file 2).

### Univariate and multivariate analysis

GGT levels and the cumulative doses of leflunomide and prednisolone were significantly and positively associated with LSM values on univariate analysis (*β *= 0.249, *β *= 0.285 and *β *= 0.36, respectively) (Table 2). On multivariate logistic regression of these significant variables, the cumulative dose of leflunomide was the only predictor of abnormal LSM values (*P *= 0.007) (Table 3).

**Table 2:** Univariate analysis of association between liver stiffness measurement (LSM) and other variables

	Beta	95% Confidentialinterval	*P*-value
Demographic variables			
Age, years	- 0.145	- 0.054, 0.008	0.140
Body mass index, kg/m^2^	0.134	- 0.041, 0.227	0.173
Disease duration, weeks	0.090	- 0.001, 0.004	0.362
Laboratory variables			
C-reactive protein, mg/L	0.051	- 0.019, 0.032	0.608
Erythrocyte sedimentation rate, mm/hr	- 0.147	- 0.026, 0.004	0.135
White blood cell, count/mm^3^	- 0.071	0.000, 0.000	0.473
Hemoglobin, g/dL	0.050	- 0.232, 0.391	0.612
Platelet count, × 1,000/mm^3^	- 0.044	- 0.007, 0.004	0.654
Prothrombin time, INR	- 0.056	- 6.729, 3.738	0.572
Glucose, mg/dL	0.001	- 0.034, 0.034	0.995
Blood urea nitrogen, mg/dL	- 0.045	- 0.085, 0.053	0.651
Creatinine, mg/dL	- 0.061	- 2.605, 1.358	0.534
Uric acid. mg/dL	0.148	- 0.058, 0.438	0.132
Aspartate aminotransferase, IU/L	0.146	- 0.014, 0.099	0.136
Alanine aminotransferase, IU/L	0.131	- 0.014, 0.071	0.183
Total protein, mg/dL	0.002	- 0.807, 0.822	0.985
Serum albumin, mg/dL	0.089	- 0.645, 1.741	0.364
Total bilirubin, mg/dL	- 0.048	- 2.135, 1.299	0.630
Alkaline phosphatase, IU/L	- 0.372	- 0.024, 0.017	0.711
Gamma-glutamyltranspeptidase, IU/L	0.249	0.008, 0.058	0.010
Triglyceride, mg/dL	- 0.033	- 0.007, 0.005	0.738
High density cholesterol, mg/dL	- 0.185	- 0.050, - 0.001	0.059
Low density cholesterol, mg/dL	- 0.161	- 0.022, 0.002	0.101
Cumulative dose of medication, mg			
Methotrexate (105 patients)	0.108	0.000, 0.000	0.273
Leflunomide (53 patients)	0.285	0.000, 0.000	0.038
Sulfasalazine (82 patients)	0.031	0.000, 0.000	0.782
Hydroxychloroquine (65 patients)	0.072	0.000, 0.000	0.567
Prednisolone (89 patients)	0.362	0.000, 0.000	< 0.001
Meloxicam (90 patients)	- 0.012	0.000, 0.000	0.909
Celecoxib (70 patients)	0.182	0.000, 0.000	0.131

INR, international normalized ratio.

**Table 3:** Multivariate analysis of independent prediction of silent liver fibrosis

	Odds ratio	95% Confidentialinterval	*P*-value
Laboratory variables			
Gamma-glutamyltranspeptidase, IU/L	1.009	0.953, 1.068	0.757
Cumulative dose of medications, mg			
Leflunomide (53 patients)	1.000	1.000, 1.000	0.007
Prednisolone (89 patients)	1.000	1.000, 1.000	0.547

### Optimal cutoff for the cumulative dose of leflunomide in predicting abnormal LSM values

Since the cumulative dose of leflunomide was the only variable that independently discriminated patients with abnormal LSM values receiving MTX over 24 weeks, we calculated the optimal cutoff for the cumulative dose of leflunomide in predicting abnormal LSM values in 53 RA patients receiving MTX plus leflunomide, based on ROC curve analysis. We found that 19,170 mg of the cumulative dose of leflunomide was a strong predictor of abnormal LSM (AUROC 0.735, 95% confidence interval 0.568, 0.903, *P *= 0.008, sensitivity 60%, specificity 89.5%).

**Figure 2:**
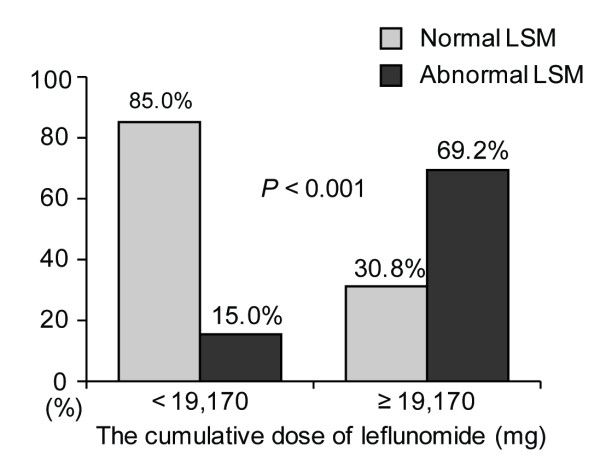
**The prevalence of abnormal liver stiffness measurement (LSM) values among patients with rheumatoid arthritis receiving methotrexate with a cumulative dose of leflunomide over 19,170 mg**. Abnormal LSM values were more frequently identified in patients with cumulative doses of leflunomide ≥ 19,170 mg than in those with cumulative doses of leflunomide < 19,170 mg (69.2% (9/13 patients) vs. 15.0% (6/40 patients), *P *< 0.001).

When we classified RA patients receiving MTX and leflunomide into two groups based on the calculated cutoff for leflunomide (19,170 mg), 13 of 53 patients were partitioned into the group with a cumulative dose of leflunomide ≥ 19,170 mg. Abnormal LSM values were identified more frequently in these patients than in those with a cumulative dose of leflunomide < 19,170 mg (69.2% (9/13 patients) vs. 15.0% (6/40 patients), *P *< 0.001) (Figure 2). Furthermore, patients with a cumulative dose of leflunomide over 19,170 mg had a significantly higher risk of having abnormal LSM values than those without (OR 12.750, *P *< 0.001, 95% confidence interval 2.952, 55.067).

## Discussion

In clinical practice, if the results of liver-related laboratory tests are abnormal, the potential liver damage and progression of fibrosis can easily be spotted by physicians, and the drug regimen or treatment strategy can be altered. However, when patients with RA have no evidence of underlying chronic liver disease and have persistently normal liver-related laboratory results, the silent progression of liver fibrosis can be missed. Indeed, data are scarce on the prevalence of silent liver fibrosis, and on how to monitor or detect this adverse outcome due to long-term use of DMARDs by patients with RA and normal liver function. Furthermore, no significant relationship between Roenigk grading of liver fibrosis and quantitative results of liver-related laboratory tests was identified in a previous study of liver biopsies performed in 16 patients with RA and long-term use of MTX [18]. Thus, in this cross-sectional study, we focused only on patients with RA who were not suspected of having underlying chronic liver disease, and found that leflunomide combined with MTX had a significant correlation with silent liver fibrosis.

So far, in addition to age, alcohol consumption, duration of RA, serum albumin level, obesity, and pre-existing pulmonary fibrosis, the cumulative dose and duration of MTX use have been reported as risk factors for histological fibrosis or cirrhosis in patients with RA receiving MTX [19-21]. Therefore, concerns related to hepatotoxicity of MTX, including elevated liver enzymes or progression of fibrosis, has limited physicians' use of MTX in RA patients with viral hepatitis or liver cirrhosis [22]. Despite these reports, in this study, we found that the cumulative dose of MTX did not significantly correlate with LSM values, and was statistically equivalent between patients with normal and abnormal LSM values, similar to the results of previous studies [13,14]. Further, when we stratified our study population into two groups based on a previously proposed cutoff MTX cumulative dose of 4,000 mg [13], the proportion of patients with abnormal LSM values was equivalent between the groups (*P *= 0.572). Also, when we reanalyzed 52 RA patients receiving MTX but not leflunomide, we found that 9 patients (17.3%) had abnormal LSM values and there were no significant differences between patients with and without abnormal LSM values. However, these results did not suggest that the cumulative dose of MTX may not be related to silent liver fibrosis in RA patients. Our study design, based on the study of patients with normal liver function, without underlying chronic liver disease, and who had been exposed to MTX for more than 24 weeks, might have reduced the extent of the effect of MTX. To clarify this, a further study will be necessary, including RA patients regardless of the administration of MTX.

Meanwhile, leflunomide has been reported to increase the frequency of abnormal liver enzyme up to 19% [23]. The currently recommended monitoring guidelines suggest leflunomide dose reduction or discontinuation when ALT levels are more than two to three times the normal level [24]. However, no reports are available that propose practical guidelines for monitoring leflunomide hepatotoxicity in RA patients with normal liver function. In our study, we found that the cumulative dose of leflunomide correlated closely with LSM values, and could be used as an independent predictor for abnormal LSM values. We selected 53 patients (51%) who received both leflunomide and MTX, and obtained the cutoff for the leflunomide cumulative dose of 19,170 mg (5.3 years with 10 mg tablets or 2.6 years with 20 mg tablets), because in Korea, leflunomide is usually administered with MTX [25]. Patients with a cumulative dose of leflunomide over 19,170 mg had a significantly higher risk of having an abnormal LSM value than those with less than 19,170 mg; the hazard ratio was 12.75. On the other hand, when we divided patients into two groups according to the presence of leflunomide and compared LSM values between the two groups, patients receiving MTX plus leflunomide (*n *= 53) had higher LSM values than those receiving MTX only (*n *= 52) (5.0 ± 2.2 vs 4.3 ± 0.9, *P *= 0.035). However, we found no significant difference in the frequency of abnormal LSM values according to the presence of leflunomide (*P *= 0.134). Thus, in RA patients receiving MTX and a cumulative dose of leflunomide over 19,170 mg (rather than the fact of its administration), we suggest that transient elastography be performed to for check silent liver fibrosis, even if the patient has normal liver function.

In our study, silent liver fibrosis was assessed using noninvasive transient elastography, instead of invasive liver biopsy. We defined 5.3 kPa as the cutoff for abnormal LSM values, which was adopted from a previous study that investigated the normal range of LSM values in healthy living liver and kidney donors in South Korea (5^th ^- 95^th ^percentiles for LSM 3.9 to 5.3 kPa) [10]. Although our patients had normal liver function and were without chronic liver disease, their range of LSM values seemed slightly higher (5^th ^to 95^th ^percentiles 3.2 to 6.7 kPa) than healthy Koreans, potentially because of long-term use of DMARDs. A value of 5.3 kPa in our study seems relatively low to predict the presence of clinically significant liver fibrosis. However, this strict cutoff may draw physicians' attention to silent liver fibrosis in patients with RA receiving MTX and leflunomide, and encourage the adjustment of hepatotoxic medication doses to prevent irreversible liver fibrosis.

Our study has several issues. First, the lack of histological data is the main limitation, especially in patients with abnormal LSM values. Second, the cutoff LSM value of 5.3 kPa is not high enough to analyze the prevalence of clinically significant fibrosis [26]. Further study with higher prevalence of high LSM values can overcome this issue. Third, because this study was cross-sectional, baseline LSM values prior to the initiation of DMARDs were not available. Finally, serial measurements of LSM for monitoring changes in the fibrotic burden were not available, in spite of its noninvasiveness. If future studies can serially measure LSM, they might reveal a dynamic correlation between LSM and differing doses of DMARDs, including MTX and leflunomide.

## Conclusions

In this study, the cumulative dose of leflunomide correlated closely with the presence of silent liver fibrosis, reflected by abnormal LSM values, and it was the only independent predictor of abnormal LSM in patients with RA, who had received MTX over 24 weeks. However, further studies are required to investigate whether treatment regimens or strategies should be modified when abnormal LSM values are identified.

## Abbreviations

ALP: alkaline phosphatase; ALT: alanine aminotransferase; AST: aspartate aminotransferase; AUROC: area under the receiver operator characteristic curve; BMI: body mass index; DMARD: disease-modifying anti-rheumatic drug; GGT: gamma-glutamyltranspeptidase; HDL: high density lipoprotein; INR: international normalized ratio; kPa: kilopascals; LSM: liver stiffness measurement; MTX: methotrexate; NSAID: non-steroidal anti-inflammatory drug; OR: odds ratio; RA: rheumatoid arthritis; TNF: tumor necrosis factor.

## Competing interests

The authors declare that they have no competing interests.

## Authors' contributions

All authors contributed to the study concept, design, and acquisition and interpretation of data. SWL, HP, SK performed the statistical analysis. SWL, SKL, SK and YB drafted and revised the manuscript. All authors have read and approved the manuscript for publication.

## Supplementary Material

Additional file 1 **The definition of the cumulative dose of each medication**. Figure showing the cumulative dose of each medication, which was defined as the accumulated area under the curve (AUC) from the time of drug initiation to the time of liver stiffness measurement (LSM) during interval follow-up days. Click here for file

Additional file 2 **Correlation between liver stiffness measurement (LSM) values and other variables**. Table showing gamma-GT levels and the cumulative doses of leflunomide and prednisolone significantly correlated with LSM; the cumulative dose of methotrexate showed no significant correlation with LSM. Click here for file
