# Supplementary figures and images for: Leflunomide increases the risk of silent liver fibrosis in patients with rheumatoid arthritis receiving methotrexate
Source: Arthritis Res Ther. 2012 Oct 29;14(5):R232. doi: 10.1186/ar4075 (PMC3580544; doi:10.1186/ar4075)

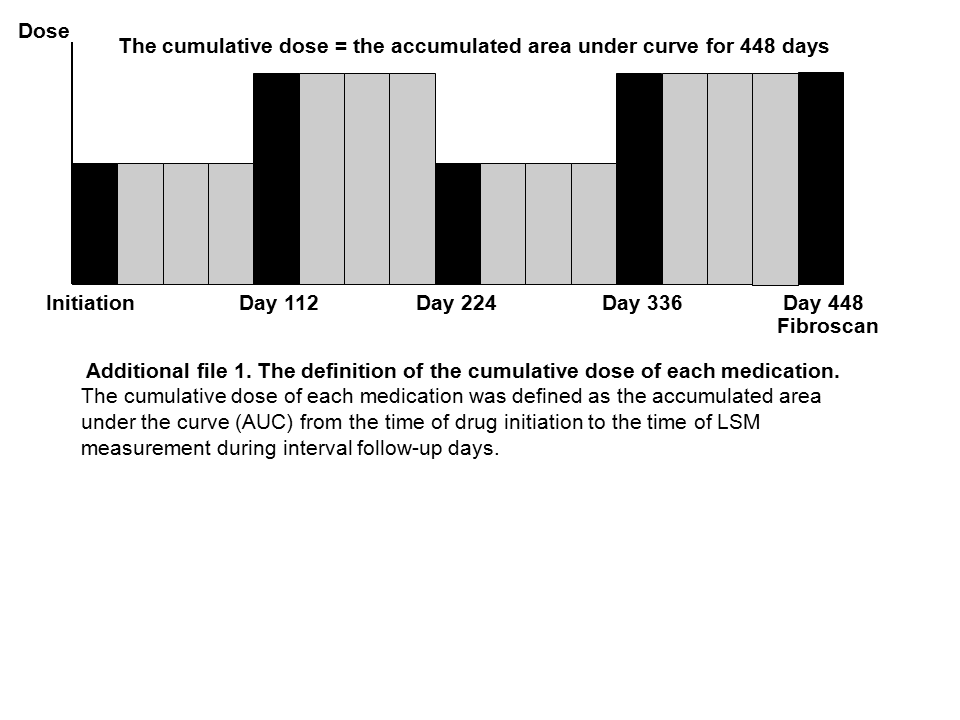

Supplement: Additional file 1 — The definition of the cumulative dose of each medication. Figure showing the cumulative dose of each medication, which was defined as the accumulated area under the curve (AUC) from the time of drug initiation to the time of liver stiffness measurement (LSM) during interval follow-up days. [file ar4075-S1.TIFF]
