# Supplementary material for: Leflunomide increases the risk of silent liver fibrosis in patients with rheumatoid arthritis receiving methotrexate
Source: Arthritis Res Ther. 2012 Oct 29;14(5):R232. doi: 10.1186/ar4075 (PMC3580544; doi:10.1186/ar4075)
Supplement: Additional file 2 — Correlation between liver stiffness measurement (LSM) values and other variables. Table showing gamma-GT levels and the cumulative doses of leflunomide and prednisolone significantly correlated with LSM; the cumulative dose of methotrexate showed no significant correlation with LSM. [file ar4075-S2.PDF]

Additional file 2. Correlation between LSM values and other variables

	Correlation coefficient	P value
**Demographic variables**		
Age (years)	-0.145	0.140
Male gender		
Body mass index (kg/m^2)	0.134	0.173
Metabolic syndrome		
**Disease duration (weeks)**	0.090	0.362
**Laboratory variables**		
C-reactive protein (mg/L)	0.051	0.608
Erythrocyte sedimentation rate (mm/hr)	-0.147	0.135
White blood cell (/mm^3)	-0.071	0.473
Hemoglobin (g/dL)	0.050	0.612
Platelet count (x1,000/mm^3)	-0.044	0.654
Prothrombin time (INR)	0.017	0.864
Glucose (mg/dL)	0.001	0.995
Blood urea nitrogen (mg/dL)	-0.045	0.651
Creatinine (mg/dL)	-0.061	0.534
Uric acid (mg/dL)	0.148	0.132
Aspartate aminotransferase (IU/L)	0.146	0.136
Alanine aminotransferase (IU/L)	0.131	0.183
Total protein (mg/dL)	0.002	0.985
Serum albumin (mg/dL)	0.059	0.548
Total bilirubin (mg/dL)	-0.048	0.630
Alkaline phosphatase (IU/L)	-0.037	0.711
Gamma-glutamyltranspeptidase (IU/L)	0.249	**0.010**
Triglyceride (mg/dL)	-0.033	0.738
High density cholesterol (mg/dL)	-0.185	0.059
Low density cholesterol (mg/dL)	-0.161	0.101

**Cumulative dose of medications (mg)**		
Methotrexate (n=105)	0.108	0.273
Leflunomide (n=53)	0.285	**0.038**
Sulfasalazine (n=82)	0.031	0.782
Hydroxychloroquine (n=65)	0.072	0.567
Prednisolone (n=89)	0.362	**<0.001**
Meloxicam (n=90)	-0.012	0.909
Celecoxib (n=70)	0.182	0.131
